# Esophageal Plasmacytoma Diagnosed in a Patient Presenting with Cardiac Symptoms: A Novel Case

**DOI:** 10.1155/2013/121670

**Published:** 2013-09-10

**Authors:** Cheryl Rimmer, Anup Hazra, Vito M. Gulli, Richard Siderits, Mark Castaldi, Zafar Zamir

**Affiliations:** ^1^Robert Wood Johnson University Hospital Hamilton, Pathology Department, Hamilton, NJ 08690, USA; ^2^Robert Wood Johnson University Hospital Hamilton, Radiology Department, Hamilton, NJ 08690, USA; ^3^Robert Wood Johnson University Hospital Hamilton, Hamilton Gastroenterology, Hamilton, NJ 08690, USA

## Abstract

Extramedullary plasmacytoma is the uncommon phenomenon of a plasma cell neoplasm occurring outside of the bone marrow. Primary plasmacytoma is a rare occurrence in the gastrointestinal tract and exceptional to originate in the esophagus. We present a novel case of a 62-year-old man who presented to our emergency department with chest pain. A cardiovascular workup was negative, and an endoscopy was subsequently performed. The endoscopy findings showed evidence of Grade IV esophagitis with ulcerations extending from 25 cm to 32 cm. Histopathological examination revealed marked acute and chronic inflammation, granulation tissue, and overlying necroinflammatory exudate. However, sheets of plasma cells, some with prominent nucleoli, were also seen. Immunohistochemically, the plasma cells expressed CD138 and MUM1 and were IgG kappa restricted. A bone marrow biopsy was performed which was negative for involvement. This is a novel case of esophageal plasmacytoma diagnosed on endoscopy in a patient presenting with acute chest pain.

## 1. Case Report

A 62-year-old man presented to our emergency department due to acute onset chest pain. He originally presented three days earlier with similar symptoms but was released from the emergency department when the cardiovascular workup was negative. His current chest pains were localized to the midsubsternal area. Morphine and nitrate treatment were not effective in relieving his pain.

The patient has a history of seizure disorder, high blood pressure, reflux disease, gastritis, hiatal hernia, osteoporosis, and osteoarthritis. He had encephalitis at approximately 18 months of age and a subsequent head injury. At 3 years old, the patient had a large benign brain tumor which was removed. These early incidents resulted in developmental and physical disabilities.

On admission his troponin I level was minimally elevated, and D-dimer was elevated. The patient was anemic and an upper endoscopy was scheduled to rule out a gastrointestinal bleed.

**Figure 1:**
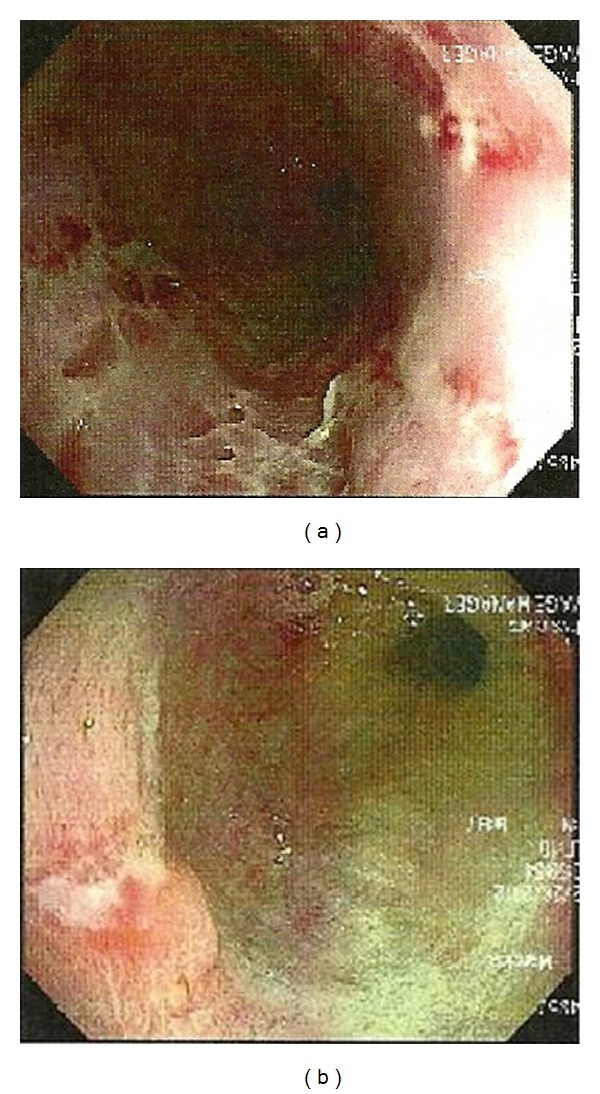
Ulcerations were seen in the esophagus (a). Ulcerations were seen in hiatal hernia (b).

The endoscopy findings showed evidence of Grade IV esophagitis. The ulcerations extended in the esophagus from 25 cm to 32 cm. A 15 mm linear ulcer was also seen on a hiatal hernia (Figure 1). The stomach and duodenum appeared normal.

**Figure 2:**
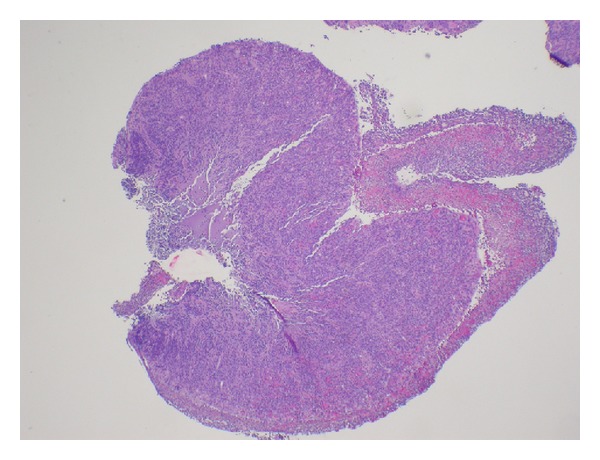
Sheets of plasma cells with the overlying esophageal mucosa showing necroinflammatory exudate.

**Figure 3:**
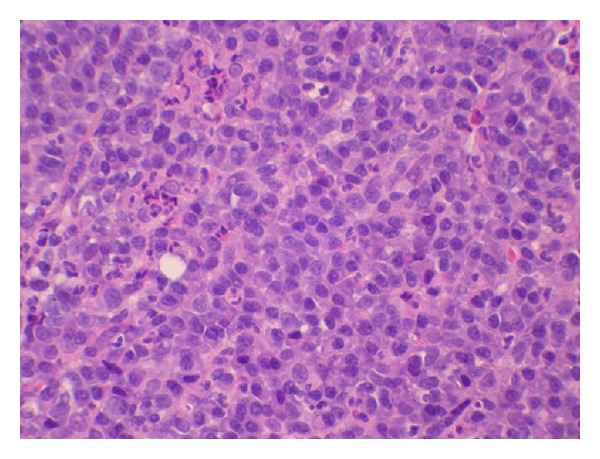
High power (40x) shows an H&E stained section with sheets of plasma cells, many with prominent nucleoli.

The biopsy results of the esophagus showed esophageal squamous mucosa with marked acute and chronic inflammation, granulation tissue, and overlying necroinflammatory exudate. In addition, there were abundant sheets of plasma cells, seen in all biopsy fragments (Figure 2). The atypical plasma cells demonstrated abundant cytoplasm with eccentrically placed nuclei, some with prominent nucleoli (Figure 3).

**Figure 4:**
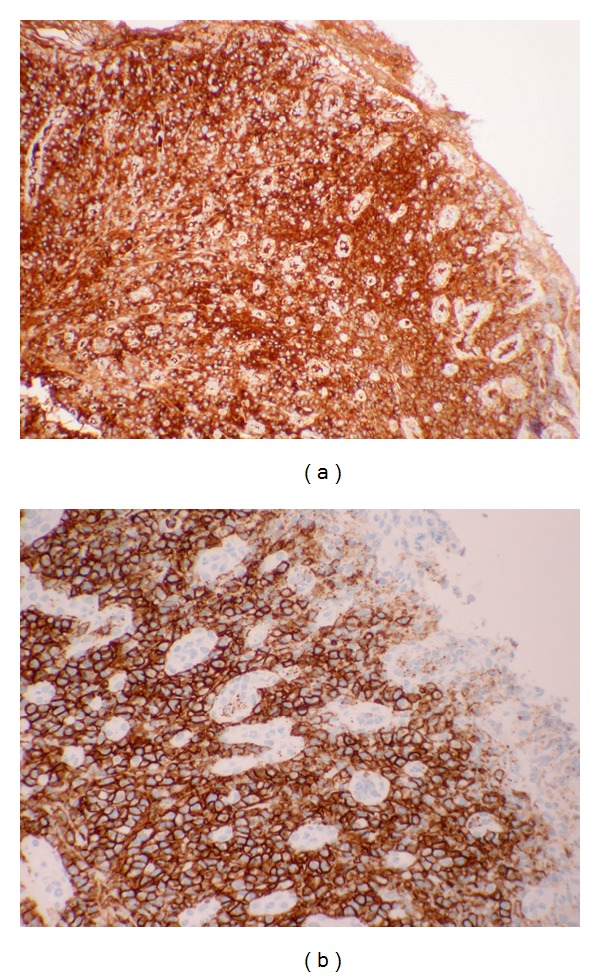
The abundant plasma cells express CD138 (a) and show kappa restriction (b).

Immunohistochemical analysis revealed that the plasma cells expressed CD138 and MUM1 and were IgG kappa restricted (Figure 4).

A whole body PET/CT scan was subsequently performed. The findings showed a focal area of increased metabolic activity in the midesophagus which may correspond to the biopsy site. An enlarged and abnormally metabolically active lymph node was identified in the left paratracheal position measuring 1.3 cm. There was also an enlarged lymph node identified in the left submental location measuring 1.1 cm. 

A bone marrow biopsy was performed which showed a  normocellular marrow with maturing trilineage hematopoiesis. There was no histologic, flow cytometric, or immunophenotypic evidence of clonal plasma cell disorder or significant plasma cell infiltrate.

**Figure 5:**
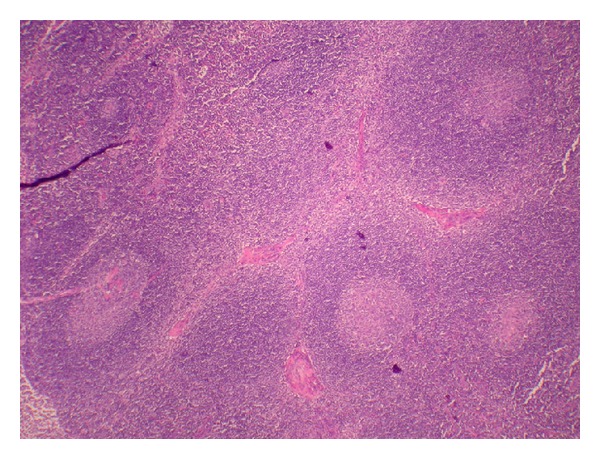
The lymph node shows well preserved architecture with slight expansion of the T zones and focal plasmacytosis.

**Figure 6:**
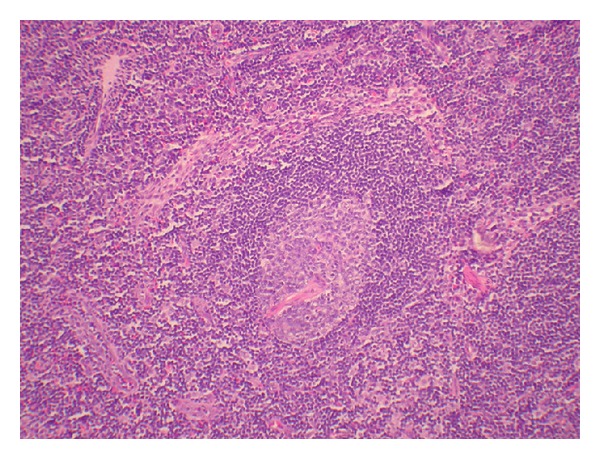
Germinal centers with penetrating blood vessels and thickening of the mantle zone, Castleman-like changes, are noted.

The submental lymph node was removed two months later for histopathologic examination. The histologic evaluation revealed preserved architecture, patent subcapsular sinuses, lymphoid follicles with germinal center formation, and slight expansion of the T-zones (Figure 5). Focal areas of Castleman disease-like changes were noted (Figure 6).

Immunohistochemical stains demonstrated normal distribution of B cell and T cells. Focal plasma cell aggregates were seen and highlighted with CD138 immunostain. There was an excess of kappa-expressing cells with cluster and aggregate formation, therefore low level involvement by a plasma cell process could not be excluded.

## 2. Discussion

Multiple myeloma is a systemic disease of monoclonal plasma cells. Involvement outside of the bone marrow may occur as a localized disease without involving the bone marrow and is known as extramedullary plasmacytoma. Plasmacytomas may arise within bone or arise in the soft tissue. Solitary plasmacytoma of bone, extramedullary plasmacytoma, and multiple solitary plasmacytomas have been designated as separate entities as per the International Myeloma Working Group (2003) [1].

Solitary plasmacytomas account for 10% of plasma cell neoplasms. Seventy-five percent of plasmacytomas involve males. The most common site of extramedullary plasmacytoma is a mucosal site of the head and neck [1] most commonly oropharynx, nasopharynx, nasal cavity, and larynx [2]. An uncommon location of primary plasmacytomas is the gastrointestinal tract, which accounts for less than 5% of extramedullary plasmacytoma [2]. Although involvement of all portions of the gastrointestinal tract have been reported, the most common location is the small bowel. The stomach and large intestine follow in order of occurrence, while the least common location appears to be the esophagus.

Endoscopically plasmacytomas commonly show a protuberant mass on endoscopy [3]. The presentation in our case was rather unusual in that it revealed evidence of Grade IV esophagitis, with ulcerations extending from 25 cm to 32 cm as well as a 15 mm linear ulcer present on a hiatal hernia.

The presenting symptoms in cases of esophageal plasmacytoma commonly include dysphagia, particularly with food consumption as well as anemia and weight loss. Clinically, the symptoms may mimic typical reflux symptoms, Schatzki rings, or esophageal carcinoma. In our case, the patient presented with midsubsternal chest pain and was worked up for possible acute myocardial injury. After cardiac disease was ruled out, a CBC showed anemia, and endoscopic evaluation was performed.

Once diagnosed on esophageal biopsy, further workup was performed including a bone marrow biopsy which showed normocellular hematopoiesis. An enlarged lymph node was examined which showed reactive changes with preserved architecture, and slight expansion of the T zones was seen. Focal areas of Castleman disease-like changes were noted. There were focal small aggregates of plasma cells with an excess of kappa-expressing cells with cluster and aggregate formation.

The lymph node was diagnosed as having focal plasmacytosis with mild kappa excess with focal areas having Castleman disease-like changes. Most commonly, Castleman's disease shows a polyclonal expression of kappa and lamba. Radaszkiewicz et al. showed that seven of eighteen lymph nodes diagnosed with Castleman's disease contained a monoclonal plasma cell population, five with IgG/lambda and two with IgA/lambda. Two of the patients had generalized lymphadenopathy although none of the patients had any evidence of a plasmacytomas. The monoclonal plasma cell type of Castleman's disease was not shown to progress to generalized plasmacellular disease. It was postulated that the monoclonal variant may be benign monoclonal gammopathy seen exclusively in lymph nodes [4].

Plasmacytomas are treated with radiation therapy followed by surgical removal. Studies have shown that the addition of chemotherapy does not increase overall survival [5]. Zhou et al. reported that 85% of patients with localized plasmacytoma achieved complete remission with radiotherapy and surgery. Eleven percent attained partial remission, and four percent did not respond to therapy. Fifteen percent of patients developed multiple myeloma and their 15-year survival rate was 78% [6].

We have diagnosed a novel case of an extramedullary plasmacytoma which presented clinically as a cardiac event. Further workup demonstrated anemia, and endoscopic examination revealed an associated Grade IV ulcerative esophagitis. Ultimately, immunohistochemical stains showed a clonal population of plasma cells. This is the first case of biopsy proven plasmacytoma in a patient with complaints of cardiac chest pain.
